# Transverse Anderson Localization in Disordered Glass Optical Fibers: A Review 

**DOI:** 10.3390/ma7085520

**Published:** 2014-07-28

**Authors:** Arash Mafi, Salman Karbasi, Karl W. Koch, Thomas Hawkins, John Ballato

**Affiliations:** 1 Center for High Technology Materials and the Department of Physics and Astronomy, University of New Mexico, Albuquerque, NM 87131, USA; 2 Department of Electrical Engineering and Computer Science, University of Wisconsin-Milwaukee, Milwaukee, WI 53211, USA; 3 Department of Electrical and Computer Engineering, University of California, San Diego, La Jolla, CA 92093, USA; E-Mail: karbasi2@uwm.edu; 4 Optical Physics and Networks Technology, Corning Incorporated, SP-AR-01-2, Sullivan Park, Corning, NY 14831, USA; E-Mail: kochkw@corning.com; 5 Center for Optical Materials Science and Engineering Technologies (COMSET) and the Department of Materials Science and Engineering, Clemson University, Clemson, SC 29625, USA; E-Mails: hawkin2@clemson.edu (T.H.); jballat@clemson.edu (J.B.)

**Keywords:** Anderson localization, optical fiber, random optical fiber, disordered optical fiber, nanostructured optical fiber, microstructured optical fiber, glass optical fiber, imaging fiber

## Abstract

Disordered optical fibers show novel waveguiding properties that can be used for various device applications, such as beam-multiplexed optical communications and endoscopic image transport. The strong transverse scattering from the transversely disordered optical fibers results in transversely confined beams that can freely propagate in the longitudinal direction, similar to conventional optical fibers, with the advantage that any point in the cross section of the fiber can be used for beam transport. For beam multiplexing and imaging applications, it is highly desirable to make the localized beam radius as small as possible. This requires large refractive index differences between the materials that define the random features in the disordered fiber. Here, disordered glass-air fibers are briefly reviewed, where randomly placed airholes in a glass matrix provide the sufficiently large refractive index difference of 0.5 for strong random transverse scattering. The main future challenge for the fabrication of an optimally disordered glass-air fibers is to increase the fill-fraction of airholes to nearly 50% for maximum beam confinement.

## 1. Introduction

Anderson localization is the absence of diffusive wave transport in highly disordered scattering media [1,2,3]. Its origin dates back to a theoretical study conducted by Anderson [1] who investigated the behavior of spin diffusion and electronic conduction in random lattices. It was soon realized that because the novel localization phenomenon is due to the wave nature of the quantum mechanical electrons scattering in a disordered lattice, it can also be observed in other coherent wave systems, including classical ones [4,5,6,7]. 

The fact that Anderson localization was deemed possible in non-electronic systems was encouraging, given that the observation of disorder-induced localization was shown to be inhibited by thermal fluctuations and nonlinear effects in electronic systems. Over the next few years, localization was studied in various classical systems including in acoustics, elastics, electromagnetics, and optics [6,8,9,10]. It was also recently investigated in various quantum optical systems, such as atomic lattices [11] and propagation of quantum mechanical photons [12]. 

General studies of Anderson localization have revealed that waves in one-and two-dimensional unbounded disordered systems are always localized. However, in order for a three-dimensional (3D) random wave system to localize, the scattering strength needs to be larger than a threshold value. This statement is often cast in the form of *kl^∗^* ∼ 1, where *k* is the effective wavevector in the medium, and *l^∗^* is the wave scattering transport length. This is referred to as the Ioffe-Regel condition [13] and shows that in order to observe Anderson localization, the disorder must be strong enough such that the wave scattering transport length becomes of the order of the wavelength. The Ioffe-Regel condition is notoriously difficult to satisfy in 3D disordered-media wave systems. For example, for the optical field to localize in 3D, very large refractive index contrasts are required that are not generally available in low-loss optical materials [6]. The fact that Anderson localization is hard to achieve in 3D optical systems may be a blessing in disguise; otherwise, no sunlight would reach the earth on highly cloudy days. 

Among all classical wave systems, optics has a unique place for the observation of Anderson localization. Optical phenomena are easy to understand and “visualize”, there are many good characterization tools and techniques available in optics, and there are potentials for device-level application of the localization phenomena in the optical domain [14,15,16]. 

**Figure 1:**
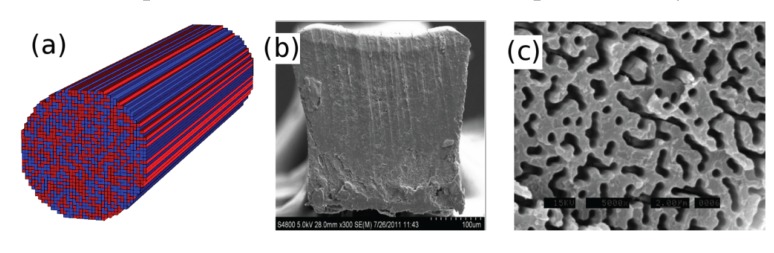
(**a**) Sketch of the transversely random and longitudinally invariant dielectric medium for the observation of the transverse Anderson localization of light; (**b**) cross section of polymer Anderson localized fiber (pALOF) with a nearly square profile and an approximate side width of 250 µm; and (**c**) zoomed-in scanning electron microscope (SEM) image of a 24 µm wide region on the tip of pALOF exposed to a solvent to differentiate between polymethyl methacrylate (PMMA) and polystyrene (PS) polymer components, where feature sizes are around 0.9 µm. Darker regions are PMMA. Reprinted/Reproduced with permission from Optics Letters, 2012 [14], and the Optical Society of America.

Unlike 3D lightwave systems in which the observation of the localization is prohibitively difficult, observation of Anderson localization in a quasi-2D optical system is readily possible, as was first shown by Abdullaev *et al*. [17] and De Raedt *et al.* [18]. In particular, De Raedt *et al.* [18] showed that two-dimensional (2D) Anderson localization can be observed in a dielectric with transversely random and longitudinally uniform refractive index profile (see Figure 1a). An optical field that is launched in the longitudinal direction tends to remain localized in the transverse plane as it propagates in the longitudinal direction in the transversely random dielectric medium. This behavior was dubbed transverse localization of light and was experimentally confirmed by Segev’s team in 2007 [19]. 

In their pioneering work, Schwartz *et al*. [19] wrote the transversely disordered and longitudinally invariant refractive index profiles in a photorefractive crystal using a laser beam. They used another probe beam to investigate the transverse localization behavior. Their experiment was quite interesting as it allowed them to vary the disorder level by controlling the laser illumination of the photorefractive crystal in a controlled fashion to observe the onset of the transverse localization and the change in the localization radius as a function of the disorder level. The transverse localization of the beam and the free longitudinal propagation due to the longitudinal invariance of the dielectric medium strongly resembled the optical waveguides; therefore, applications of Anderson localization for light propagation in an optical fiber-like medium seemed an appealing extension of these ideas. However, the variations of the refractive index of random sites in Reference [19] were of the order of 10^−^^4^. Such small variations in the refractive index of random dielectric result in a very large mean value and standard deviation of the localized beam radius. In order to obtain an optical fiber-like behavior with a localization radius comparable to that of conventional optical fibers, large refractive index fluctuations are required. This was addressed first by Karbasi *et al.* [14] in a disordered polymer fiber in 2012, the details of which will be discussed in the next section. 

## 2. Disordered Polymer Optical Fiber

In order to obtain large refractive index fluctuations required for a small transverse localization radius, Karbasi *et al*. [14] used a polymer random optical fiber medium. The refractive index fluctuations of the structure were of the order of 0.1. The random refractive index profile of the polymer Anderson localized fiber (pALOF) was fabricated from a low index component polymethyl methacrylate (PMMA) with refractive index of 1.49 and a high index component polystyrene (PS) with refractive index of 1.59. 40,000 pieces of PMMA and 40,000 pieces of PS fibers were randomly mixed [20], fused together, and redrawn to a fiber with a nearly square profile and approximate side width of 250 µm as shown in Figure 1b. The refractive index profile remained invariant for the typical lengths used in the experiment because of the large redraw ratio [21]. Figure 1c shows the zoomed-in scanning electron microscope (SEM) image of a 24 µm wide region on the tip of pALOF after exposing the tip to ethanol solvent to dissolve the PMMA, where the typical random feature size is around 0.9 µm. 

The large index contrast of 0.1 ensures that the localization radius is sufficiently small and comparable to that of conventional optical fibers. Moreover, the sample-to-sample fluctuation over the ensemble of localized beam radii has been shown to be smaller for larger index contrast. Therefore, the localized beam radii launched at different transverse positions over the facet of pALOF are all small and nearly identical. We note that the smallest possible sample-to-sample variation is desired for device-level applications of the waveguides using the transverse Anderson localization mechanism, otherwise the beam radius can vary considerably across the fiber and also among different fibers. We also emphasize that pALOF does not have a core/cladding structure; therefore, the entire transverse cross section of the fiber can be used to transport light, unlike the conventional optical fibers where light is only guided by the core. This property can be used in applications were beam spatial multiplexing is needed, for example in beam-multiplexed optical communications [22] and optical imaging [16]. A successful attempt in using pALOF for imaging applications is described in the next section. 

## 3. Random Fiber for Imaging

One of the most attractive properties of Anderson-localized optical fibers is that they can be used to transport transversely localized beams coupled to any location across their transverse profile. Moreover, the localized beams are shown to have very low cross-talk and extremely low bend loss values [15]. Therefore, they can be used for the simultaneous propagation of multiple spatially separated beams, and especially for direct image transport [23]. Recently, pALOF was successfully used for endoscopic fiber-optic imaging and the image transport quality was shown to be comparable with or better than some of the best commercially available multicore image fibers, with less pixelation and higher contrast [16]. 

**Figure 2:**
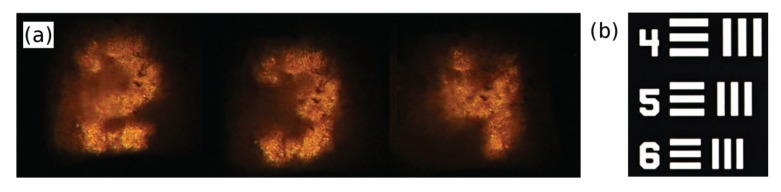
(**a**) Transported images of different numbers through 5 cm of pALOF are shown; and (**b**) a section of the 1951 U.S. Air Force resolution test chart (1951-AFTT) used in the image transport experiment is shown. Details can be obtained in [16].

Figure 2 shows some of the transported images in the form of numbers from a section of the 1951 U.S. Air Force resolution test chart (1951-AFTT) through a 5 cm-long pALOF. The test-target in the form of a stencil in which numbers and lines were carved was butt-coupled to a hand-polished input facet of pALOF and was illuminated by white light. The near-field output was projected onto a charge-coupled device (CCD) camera with 40× and 60× microscope objectives. 

The main limiting factors of the image transport quality in pALOF include the cleaving and polishing quality of the end facets. Moreover, the maximum good-quality image transport length has been limited to 16 cm. The image transport length is expected to increase significantly by using better fabrication techniques in a clean-room environment. An improved fiber drawing process is also required to ensure that the fiber radius remains invariant and does not vary more than a few percent over the desired imaging length (one meter or more), in order to ensure that the refractive profile remains largely invariant. 

The limitations mentioned above can potentially be overcome in pALOF for improved image transport. However, the ultimate limiting factor in the image transport quality is the point spread function in the imaging system that is directly related to the localization radius. Therefore, for high-quality imaging, the smallest possible localization radius is required. Among the possible design parameters that affect the localization radius, refractive index contrast plays an important role, as emphasized earlier. As a solution, the disordered optical fiber can be made from a glass matrix with random airholes. In other words, the binary disordered material in pALOF can be replaced by air and glass with the improved (and sufficiently large [24]) refractive index contrast of 0.5. As mentioned earlier, the large refractive index contrast reduces the variation in the localized beam radius across the fiber as well [21,24,25,26]; therefore, a better image uniformity can be obtained using the glass-air disordered matrix [16]. In the next section, successful attempts in observing transverse Anderson localization in random glass-air matrices are reviewed, as the first steps towards the realization of a high-quality disordered imaging fiber. 

## 4. Disordered Glass Optical Fibers

The image transport resolution through an Anderson localized fiber is limited by the beam localization radius. The localization radius can be lowered by increasing the refractive index difference between the materials that define the random features in the disordered fiber. This is intuitively understandable, given that the higher index contrast results in stronger transverse scattering and ultimately a stronger transverse localization. Another parameter that controls the localization radius in the glass-air matrix is the airhole fill-fraction. The ideal value in this binary disordered system is 50%, which results in the strongest transverse scattering and the smallest localization radius [21]. 

**Figure 3:**
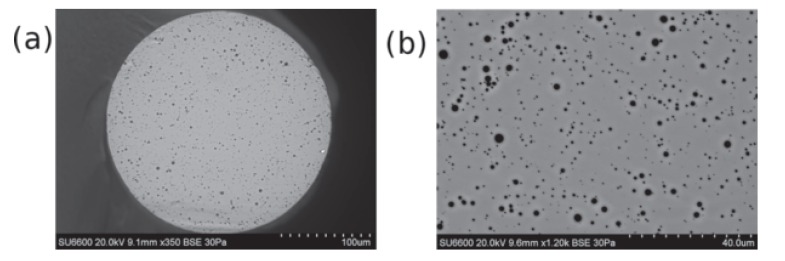
(**a**) SEM image of the glass optical fiber with random airholes reported in [27]; and (**b**) zoomed-in SEM image of the same fiber. Reprinted/Reproduced with permission from Optical Material Express, 2012 [27], and the Optical Society of America.

The first observation of Anderson localization in a silica fiber was reported by Karbasi *et al*. [27] in 2012. The reported glass-air disordered fiber was drawn at Clemson University. The preform was made from “satin quartz” (Heraeus Quartz), which is a porous artisan glass. The cross-sectional SEM image of the disordered glass-air optical fiber is shown in Figure 3a and a zoomed-in SEM image is shown in Figure 3b. The SEM images provide a good estimate of the refractive index profile of the fiber; the light gray background matrix is glass and the black random dots represent the airholes. The diameter of the disordered glass fiber is measured to be 250 µm. The diameter of the airholes vary between 0.2 µm and 5.5 µm. 

Strong localization was observed near the boundary of the glass-air random fiber in Figure 3; however, no localization was observed near the center of the fiber. The main reason behind this behavior was reported to be the non-uniform distribution of the airholes across the disordered fiber. The airhole fill-fraction was shown to be as low as 2% in the central regions, but it reached almost as high as 8% near the boundary of the fiber. While either value is substantially lower than the ideal value of 50%, the higher level of disorder near the boundary is sufficient to induce localization, certainly assisted by the large refracted index contrast of 0.5. Recently, Anderson localization with airhole fill-fraction lower than that of Reference [27] has been reported by Reference [28]; the observation of localization is attributed to the smaller air line diameter. 

The main challenge in lowering the beam localization radius in an glass-air fiber form high-quality image transport is to obtain an airhole fill-fraction of 50% as required for maximum transverse scattering [27]. Fabrication of an ideal glass-air disordered fiber using stack-and-draw technique is only conceivable if several stages of restacking/redrawing are implemented, in order to obtain the required random feature size, comparable to the reported pALOF. Assuming a total count of 90,000 for the number of random refractive index variation sites (pALOF has 80,000 random sites), a two-stage restacking of 300 fibers is required to obtain a quasi-random profile, which is practically conceivable. We note that image transport has been investigated previously in random phase-separated glasses as well [29,30]. 

## 5. Conclusions

Disordered optical fibers demonstrate many novel physical properties, mainly driven by the possibility of localized beam transport over the entire cross section of the optical fiber. Therefore, highly localized beams are typically desired in these fibers and the fiber should be designed as such. One of the main parameters that control the localized beam radius is the refractive index difference between the random features in the disordered fiber. Another important parameter that affects the localized beam radius is the relative ratio of the random binary materials, which must be close to unity (50% fill-fraction) for maximum transverse scattering to obtain the smallest possible beam radius. Disordered glass optical fibers with random airholes are highly desirable as they provide a large refractive index difference of 0.5. However, the airhole fill-fraction in the glass matrix is lower than 10% in the reported experiments, which is far below the desired value of 50%. Future efforts will concentrate on increasing the airhole fill fraction, as well as determining the optimum random feature size and distribution for high-quality image transport applications. 
